# Unforeseen complications: a case of dengue shock syndrome presenting with multi-organ dysfunction in a subtropical region

**DOI:** 10.1186/s41182-023-00530-y

**Published:** 2023-07-17

**Authors:** Syed Muhammad Owais, Farrukh Ansar, Muhammad Saqib, Khatira Wahid, Khalid Rashid, Hassan Mumtaz

**Affiliations:** 1 Northwest General Hospital & Research Centre, Peshawar, Pakistan; 2 Quaid e Azam International Hospital, Rawalpindi, Pakistan; 3 Khyber Medical College, Peshawar, Pakistan; 4 James Cook University Hospital, Middlesbrough, UK; 5 University of Sunderland, Sunderland, England UK; 6 Maroof International Hospital, Islamabad, Pakistan; 7 Health Services Academy, Islamabad, Pakistan

**Keywords:** Severe dengue, Tropical climates, Breast feeding, Postpartum sepsis, Pakistan, Neurological manifestations, Myocarditis, Acute renal failure, Acute liver failure, Case reports

## Abstract

**Overview:**

Dengue fever, a viral illness transmitted by the Aedes mosquito, is capable of causing a range of serious complications, including fulminant hepatic failure, renal dysfunction, encephalitis, encephalopathy, neuromuscular and ophthalmic disorders, seizures, and cardiomyopathy.

**Case description:**

This report details the case of a 30-year-old lactating woman with no notable medical history who presented to the emergency department with symptoms of high-grade fever, altered mental status, and seizures. Upon imaging, bilateral infarcts in the thalami and cerebellar hemispheres were observed, consistent with cerebellitis and dengue encephalitis.

**Patient treatment and outcome:**

The patient was admitted to the intensive care unit and received appropriate treatment. Following a critical phase and successful patient stabilization, she was transferred to a high dependency unit for a week before being discharged with recommendations for follow-up care.

**Conclusion:**

This case illustrates the broad spectrum of complications that can arise as a result of dengue infection and the importance of timely diagnosis and management in improving patient outcomes. Further investigation is required to better understand the mechanisms underlying these complications and to formulate specific guidelines for the prevention and treatment of dengue shock syndrome.

## Introduction

Dengue fever is a viral infection transmitted by the Aedes mosquito. It is caused by one of four serotypes of the dengue virus (DENV 1–4). The dengue virus belongs to the Flaviviridae family of ribonucleic acid (RNA) viruses [1]. Dengue is an endemic disease in tropical and subtropical countries, putting almost four billion people worldwide at risk. The prevalence of dengue has rapidly increased in the Southeast Asian region in recent years. It is important for people living in or traveling to areas where dengue is prevalent to take precautions to protect themselves from mosquito bites and to seek medical attention if they develop symptoms of dengue fever [2]. Dengue shock syndrome (DSS) is the most severe manifestation of dengue infection and can have a mortality rate of up to 20% if not treated appropriately. DSS is characterized by a rapid drop in blood pressure, leading to shock and organ failure. Early diagnosis and management of DSS is crucial for improving patient outcomes. It is important for healthcare providers to be aware of the signs and symptoms of DSS and to initiate prompt treatment in order to prevent complications and reduce mortality [1]. It has been suggested that there are over 350 million reported cases of dengue and 22,000 related deaths worldwide each year [3]. Generally, dengue infection is characterized by a high-grade fever accompanied by rigors, chills, body aches, and a transient macular rash. However, in rare cases, complicated dengue infection can lead to severe complications such as fulminant hepatic failure, renal dysfunction, encephalitis, encephalopathy, neuromuscular and ophthalmic disorders, seizures, and cardiomyopathy [4]. Severe hepatic involvement associated with dengue infection is very rare. According to a large retrospective cohort study from the Hospital for Tropical Disease in Thailand, the incidence of acute liver failure in symptomatic dengue patients was less than 0.5%, but it had a significant mortality rate of 66%. This highlights the importance of early diagnosis and management of dengue infection in order to prevent complications and reduce mortality [5].

## Case presentation

A 30-year-old lactating mother in subtropical South Asia with no significant past medical or surgical history presented to the emergency room with chief complaints of high-grade fever, altered mental status, and seizure. High grade and intermittent fever had been present since five days prior to admission, accompanied by rigors and chills. The patient’s mental status altered gradually starting with a loss of orientation and progressing to complete obtundation. The patient also experienced abrupt localized seizure in her lower limbs every half to one hour, without generalized tonic–clonic seizures or tongue bites. The patient did not have any bowel or bladder incontinence.

Physical examination revealed body temperature of 101 ºF, blood pressure of 99/64 mmHg, pulse of 144/min, oxygen saturation of 94% on room air, a respiratory rate of 36/min and a Glasgow Coma Scale score of 5/15 with a fixed constricted pupil. A malar rash on the face, palmar erythema, left lower extremity focal seizures, prolonged capillary refill, cold, clammy, and mottled skin were observed. The rest of the physical examination was unremarkable. The patient's random blood glucose was 180 mg/dl, and there were no signs of meningismus. Blood test revealed a hemoglobin level of 12.7 g/dL, a platelet count of 105 × 10^9^/L, and neutrophils of 27.5 × 10^9^/L. The alanine transaminase was 1394 U/L, C-reactive protein was 19.2 mg/dL, creatinine was 1.79 mg/dL, activated partial thromboplastin time was 61.7 s, procalcitonin was 0.00835 mg/dl, and Troponin I was raised at 0.00012168 mg/dl.

An echocardiography report showed an ejection fraction of 35–39% with mild pulmonary hypertension and moderate left ventricular systolic dysfunction. A brain computed tomography (CT) scan showed hypodensity in both the thalami and cerebellar hemispheres, suggesting bilateral thalamic and cerebellar infarcts and a possibility of cerebellitis and encephalitis. Grey–white matter differentiation appeared intact, and there was no evidence of a focal mass, midline shift, or hematoma. A brain magnetic resonance imaging (MRI) showed bilateral, almost symmetrical, high signals on T2-weighted and fluid-attenuated inversion recovery images in the thalami cerebellar hemispheres and bilateral cerebral cortices, which indicated the possibility of encephalitis or postictal ischemic changes. An enhanced CT scan of the chest and abdomen showed bilateral basal atelectasis, hepatomegaly, a distended gallbladder and enlarged bilateral iliacus muscles with internal hyperdense and hypodense areas suggesting the possibility of bilateral iliacus hematomas with some liquefaction.

The patient was diagnosed as sepsis, metabolic acidosis (evident from serum bicarbonate levels of 18 mEq/L, arterial pCO2 of 29 mmHg and a pH of 7.23), respiratory distress, acute kidney injury, heart failure due to myocarditis, acute liver injury and possible brain edema. Sudden onset of high-grade fever, systemic symptoms with multiple organ failure and local endemic situation arose the possibility of dengue shock syndrome although normal platelet count and absence of petechial rashes on the body were not compatible.

**Fig. 1:**
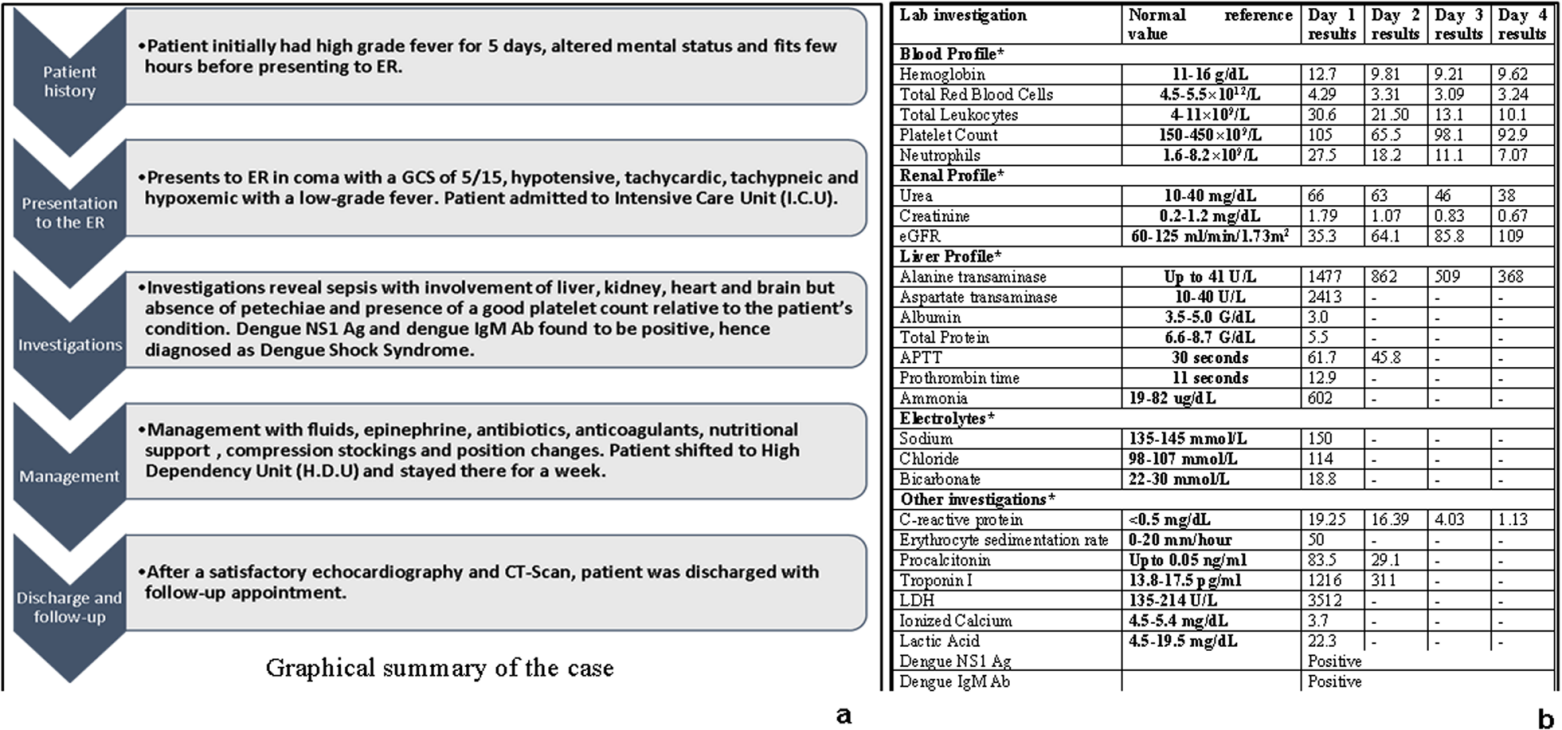
Summary of the case (**a**) and table of investigations (**b**). *Only the deranged values have been reported; Dengue NS1 Ag: dengue non-specific antigen 1; Dengue IgM Ab: dengue immunoglobulin M antibody

Further investigation revealed positive dengue non-specific antigen 1 (Dengue NS1 Ag) and positive dengue immunoglobulin M antibody (Dengue IgM Ab)done using qualitative Wondfo© One Step Dengue NS1 Antigen kits. A graphical summary of the case as well as the table of investigations can be seen in Fig. 1.

The patient was admitted to the intensive care unit and intravenous fluids were started (3% normal saline, 100 ml/h) with 0.10 μg/kg/min of norepinephrine. Mechanical ventilation was initiated due to the patient's deteriorating respiratory status, suspected secondary bacterial infection and herpes encephalitis, intravenous antibiotics (ceftriaxone 1 g/12 h and azithromycin 500 mg/day) and acyclovir (400 mg/8 h). In addition, the patient received intravenous insulin (0.1 units/kg/h) to maintain normal blood sugar levels and intravenous vasopressin (0.01 units/min) to maintain optimal blood pressure (above 120 mmHg systolic and above 80 mmHg diastolic) on the first day of admission. The patient soon started responding to treatment with gradual improvement in consciousness and laboratory findings.

The patient's renal function was monitored closely, and hemodialysis was initiated on the first day of admission. The patient's liver function was also monitored, and she received intravenous *N*-acetylcysteine and a low-fat diet. N-acetylcysteine (NAC) was administered in a specific dosing regimen. Initially, a bolus dose of 150 mg/kg body weight was administered, followed by a maintenance dosage of 12.5 mg/kg/h over a duration of 4 h. Subsequently, the maintenance dosage was adjusted to 6.25 mg/kg/h and continued for up to 72 h.

**Fig. 2:**
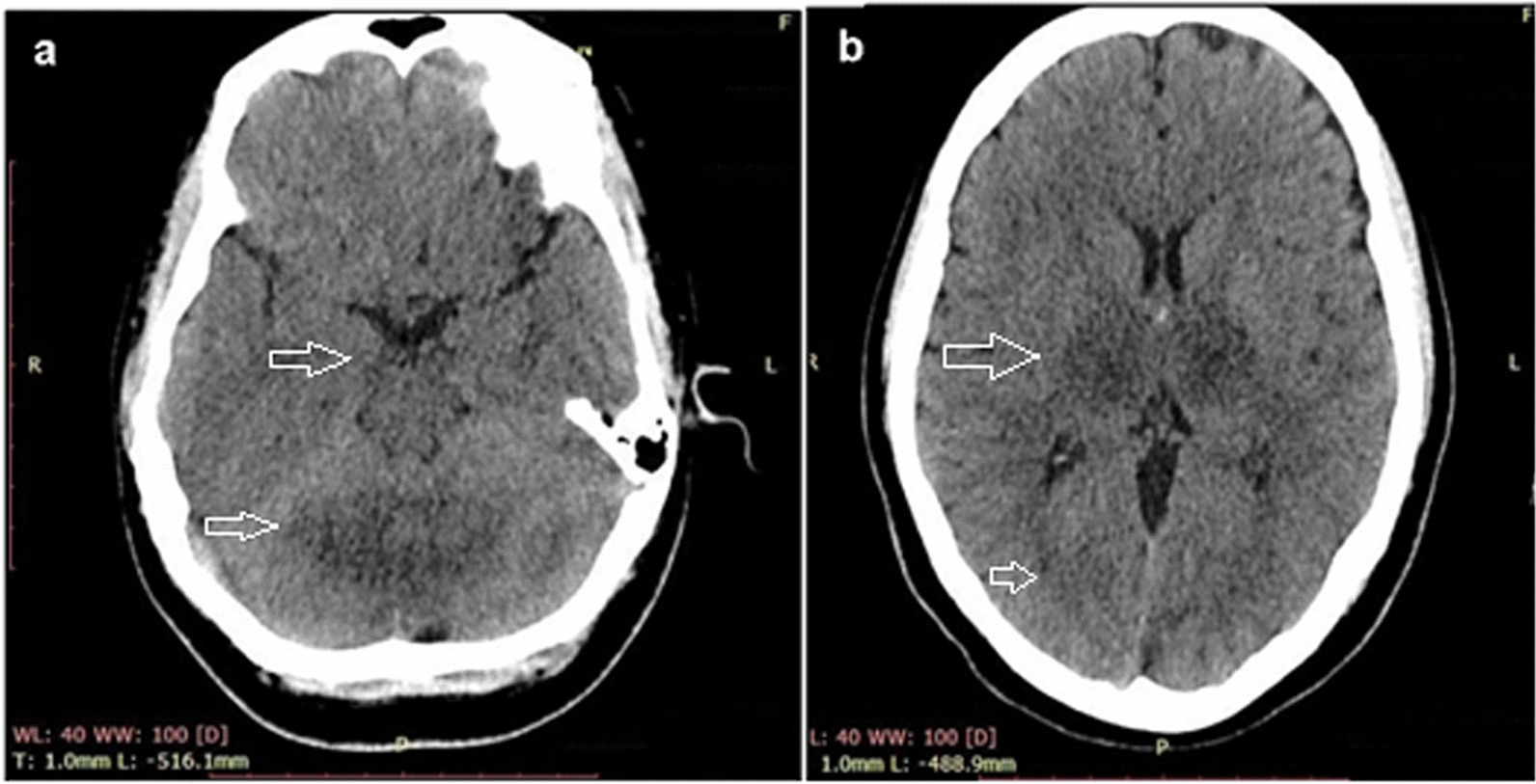
CT scan showing bilateral thalamic and cerebellar hypodensities (**a**, **b**); patient details are hidden to protect patient privacy

**Fig. 3:**
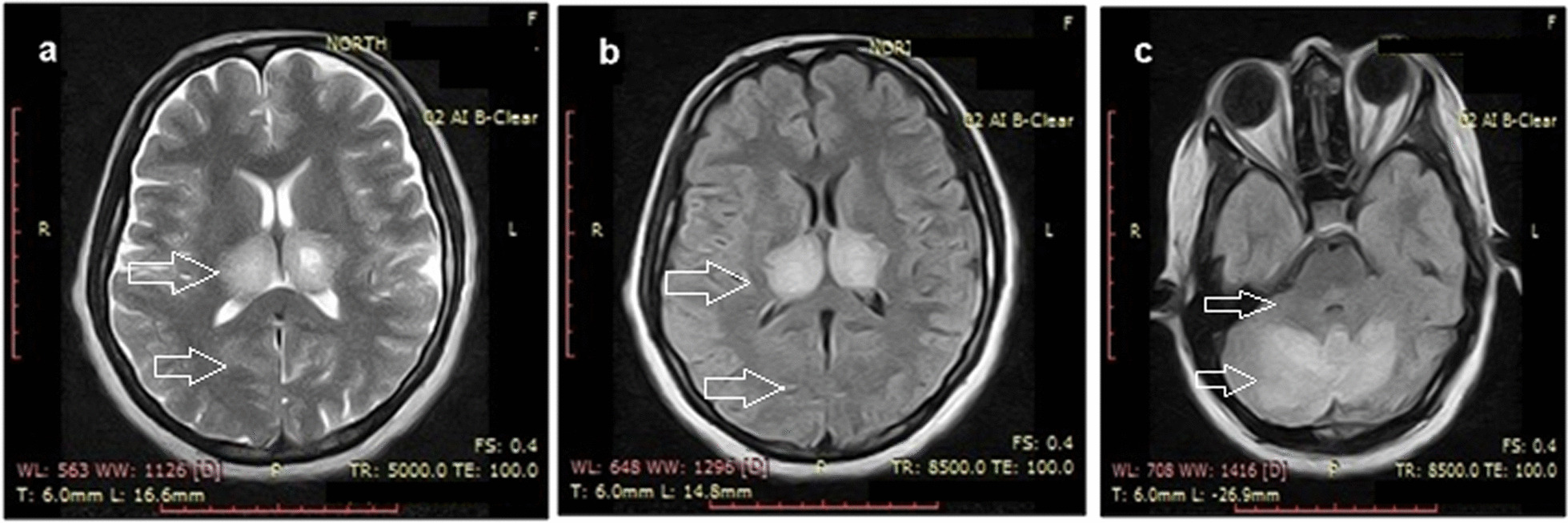
MRI scan showing bilateral thalamic and cerebellar infarcts (**a**–**c**); patient details are hidden to protect patient privacy

The patient's condition improved gradually over the next few days, and the mechanical ventilation was discontinued on the fourth day of admission. The patient was transferred to the high dependency unit for further management and stayed there for a week. After satisfactory echocardiography (revealing ejection fraction of 59% with a cardiac output 6.0 L per minute and a heart rate of 80 beats per minute, indicating a normal cardiac profile) and CT scan results (resolution of thalamic and cerebellar involvement seen on previous CT scans), the patient was discharged and advised to follow-up. CT scan and MRI images taken before recovery are shown in Figs. 2 and 3, respectively. CT scan of the brain, revealed bilateral thalamic and cerebellar infarcts, suggesting brain involvement. Additionally, a magnetic resonance imaging (MRI) of the brain showed abnormal signals in the thalami, cerebellar hemispheres, and bilateral cerebral cortices, indicating the presence of dengue encephalitis or postictal ischemic changes. These imaging findings support the diagnosis of neurological involvement in the patient.

**Fig. 4:**
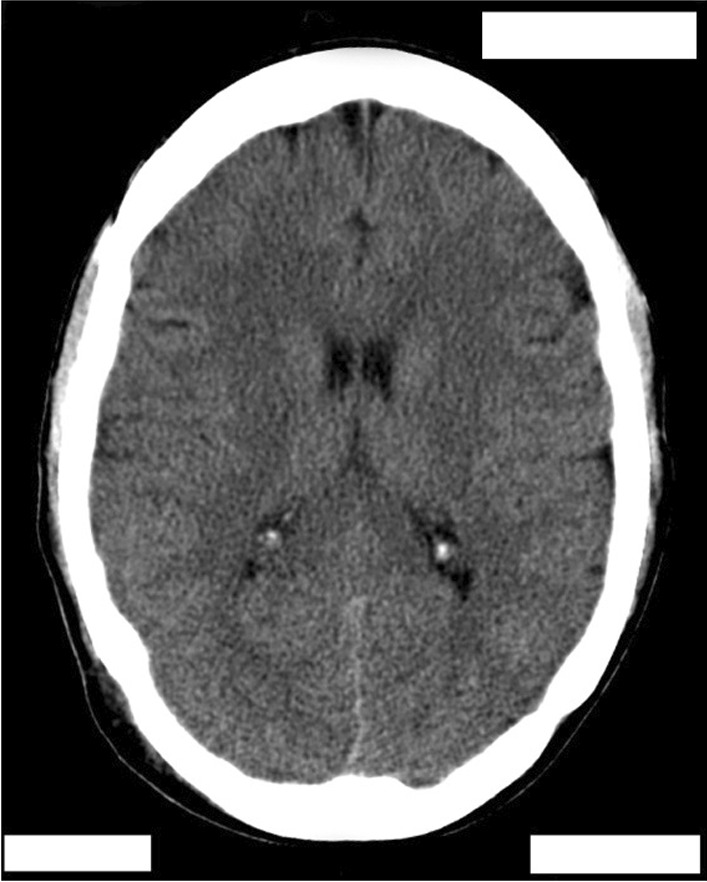
CT scan of the brain after recovery showing resolution of all findings seen on previous CT scan; patient details are hidden to protect patient privacy

The patient was conscious towards the end of day 1 and slowly improved function. There was a mild residual muscle weakness in the proximal thigh muscles which improved in the subsequent days. This could be due to the lower limb seizures that were observed in the initial phase of admission. There were no signs of muscle paralysis observed in the patient. A recovery CT scan done on day 4 showed resolution of brain findings seen on CT previously as shown in Fig. 4.

## Discussion

The relationship between dengue fever and neurological manifestations was first described in 1976, and multiple studies since then have shown that dengue fever can be associated with neurological complications [6, 7]. Neurological manifestations of dengue fever can include headaches, irritability, alteration of consciousness, insomnia, and focal neurological deficits. These manifestations may be associated with encephalitis and seizures [6]. Dengue fever presents various neurological manifestations that can be classified into three distinct categories. The first category involves direct neurotropism, leading to conditions such as encephalitis, meningitis, myelitis, and myositis. The second category encompasses systemic complications, which include encephalopathy, stroke, and hypokalemic paralysis. Lastly, there are post-infectious or immune-mediated manifestations, such as acute disseminated encephalomyelitis (ADEM), Guillain–Barré syndrome (GBS), and optic neuritis [8].

In our case, the patient belonged to a subtropical region of South Asia and presented with altered mental status, seizure, and low Glasgow Coma Scale score, which were indicative of neurological involvement. This was supported by a CT scan showing bilateral thalamic and cerebellar infarcts due to possible brain edema, possibly indicating cerebellitis and dengue encephalitis. Myocarditis and cardiac dysfunction are rare but recognized complications of dengue fever. Earlier studies have reported on these complications, but did not specify which serotype was most commonly associated with them. More recent studies, however, have suggested that dengue virus serotype 2 (DENV-2) may be particularly implicated in causing myocardial dysfunction in children. Cardiac complications of dengue fever tend to manifest early in the disease course, and common electrocardiographic changes include T-wave inversion. These findings have been described in the literature previously [9]. In the current case, our patient was suspected to have myocarditis, which was later confirmed by the presence of a raised Troponin I level and a low ejection fraction on echocardiography. Acute kidney injury (AKI) is a significant complication that can occur in patients with dengue fever, particularly in those who are hospitalized for extended periods of time. The etiology of AKI in dengue fever is not fully understood, but proposed mechanisms include rhabdomyolysis, hemodynamic instability, acute glomerular injury, and hemolysis, all of which can lead to tubular necrosis, thrombotic microangiopathy, and acute glomerulopathy. Unfortunately, patients with dengue fever who develop renal complications such as AKI have a higher mortality rate. There are currently no specific recommendations for the treatment of AKI in dengue patients, and treatment typically involves conventional renal replacement therapy [10]. Dengue fever can affect the liver, which is the most commonly affected organ in patients with this infection. Liver involvement can range from mild elevation of hepatic transaminases to severe acute liver failure. The mechanisms behind liver injury in dengue fever are not fully understood, but may include hypoxic liver injury due to shock, direct virological attack on hepatocytes, and immunological damage to the liver. The management of acute liver injury in dengue fever can be challenging, as there are few guidelines available on the best approach. In the past, some studies have suggested that the use of NAC as an antidote for acetaminophen toxicity may be beneficial in the management of acute liver failure in dengue fever, as it has been associated with reduced mortality and high transplant-free survival, particularly when used in the early stages of the disease [11]. In our case, the administration of NAC was based on evidence from a recent meta-analysis conducted by Walayat et al. [12], which highlighted the significant improvement in overall survival associated with NAC, even in cases of non-acetaminophen-related acute liver failure [12]. The underlying pathophysiology of dengue fever involves a complex interplay between the virus and host-specific factors. The dengue virus replicates inside host cells, triggering the release of immune-mediated destruction and cytokines. While there is increased vascular permeability, plasma leakage is typically confined to the pleural and peritoneal cavities and does not result in generalized edema. The development of hemorrhagic diathesis is thought to be caused by liver damage that leads to decreased secretion of coagulative factors and albumin. The virus also replicates in the adrenal gland, contributing to sodium loss and hypotension. The presence of petechiae, which are small red or purple spots on the skin, is likely due to capillary fragility, thrombocytopenia, and cytokines that disrupt vascular integrity [13, 14]. In dengue infection, both thrombosis and brain edema are potential mechanisms underlying the vascular involvement observed in cerebellitis and dengue encephalitis. Thrombosis can occur due to endothelial dysfunction and increased vascular permeability, leading to impaired blood flow and infarction in cerebral blood vessels. Meanwhile, the inflammatory response triggered by dengue fever can cause brain edema through the release of cytokines and immune mediators, resulting in increased blood–brain barrier permeability and fluid accumulation in the brain tissue. Brain edema can subsequently compress surrounding vessels and compromise blood flow, potentially leading to ischemic events and infarction [15]. It is evident from the CT images that the patient in our case most probably had ischemic changes due to brain edema that resolved in the subsequent days as evident in follow-up recovery brain CT scan which shows no residual findings.

Our patient presented to the emergency department with encephalopathy leading to coma, a neurological complication of dengue fever. There is a difference between encephalopathy and encephalitis in dengue virus infection which can be seen in Table 1.

**Table 1:** Differences between dengue encephalopathy and dengue encephalitis

Criteria	Dengue encephalopathy	Dengue encephalitis
Definition	Brain dysfunction due to the effect of dengue virus on organs other than the brain [21]	Inflammation of the brain tissue [22]
Cause	Usually secondary to derangement in multiple systems like shock, hepatitis, coagulopathy, and concurrent bacterial and viral infection [22]	Direct neuronal infiltration of the dengue virus [22]
Pathogenesis	Disruption of brain function [21]	Invasion and replication of the virus [22]
Symptoms	Altered mental status, confusion, coma [21]	Fever, headache, neurological symptoms [22]
CSF Examination	Normal or minimal abnormalities [23]	Increased protein, pleocytosis (rare)

Upon examination, the patient was found to be in shock, as indicated by tachycardia, tachypnea, hypotension, cold, clammy, and mottled skin, and prolonged capillary refill. The presence of palmar erythema and malar rash may have been due to the physiological effects of pregnancy. Initially, the absence of petechiae and a good platelet count led us to suspect a case of non-dengue viral sepsis. However, dengue antigenic testing eventually revealed a positive result. This case is unique in that it involved multiple organ involvement mimicking viral sepsis, but without evidence of petechiae and a relatively good platelet count given the patient's condition. The diagnosis of dengue infection was ultimately reached through extensive testing and an astute clinical approach.

The patient was suffering from acute liver injury, acute kidney injury (AKI), heart failure (myocarditis), hypernatremia, and possible brain edema. While previous reports have described similar complications of dengue fever, this case is unusual in that it involved all of these complications simultaneously [16–18]. Our treatment regimen was in accordance with the guidelines provided by the Centers for Disease Control and Prevention [19]. Our treatment approach was also informed by based on the findings of multiple randomized controlled trials studied by Kalayanarooj et al. [20]. In the management of our patient, we focused on restoring and maintaining intravascular volume for sufficient end-organ perfusion. To this end, we administered intravenous fluids and norepinephrine to improve hemodynamics and normalize blood pressure, as well as antibiotics to control sepsis. We did not use beta blockers to lower the patient's heart rate, but closely monitored it instead. Other treatments included oral proton pump inhibitors to prevent stress ulcers, whole-nutrition in the form of Ensure®, compression stockings to prevent deep vein thrombosis, and any other necessary medications. There are many reasons why our case is unique. First, the case presents a unique and rare combination of serious complications of dengue fever, including dengue encephalitis, suspected myocarditis, acute kidney injury, and acute liver failure. This is an unusual presentation of dengue fever that has not been widely reported in the literature and would be of interest to healthcare professionals and researchers studying this disease. Second, the case report provides a detailed account of the patient's clinical presentation, diagnostic workup, and management, including the specific treatment strategies employed to address each of the complications. This information would be valuable to other healthcare professionals caring for patients with dengue fever and could inform future clinical practice. Finally, the successful management of the patient's multiple serious complications and the patient's eventual recovery make this an informative and inspiring case report that would be of interest to a wide audience. More interdisciplinary and evidence-based studies are required to make guidelines and decide on diagnosis and optimum fluid management in dengue infections complicated by encephalopathy in lactating women with dengue infection complicated by multiple complications. The guidelines are essential to facilitate management and prevent any adverse outcomes.

**Fig. 5:**
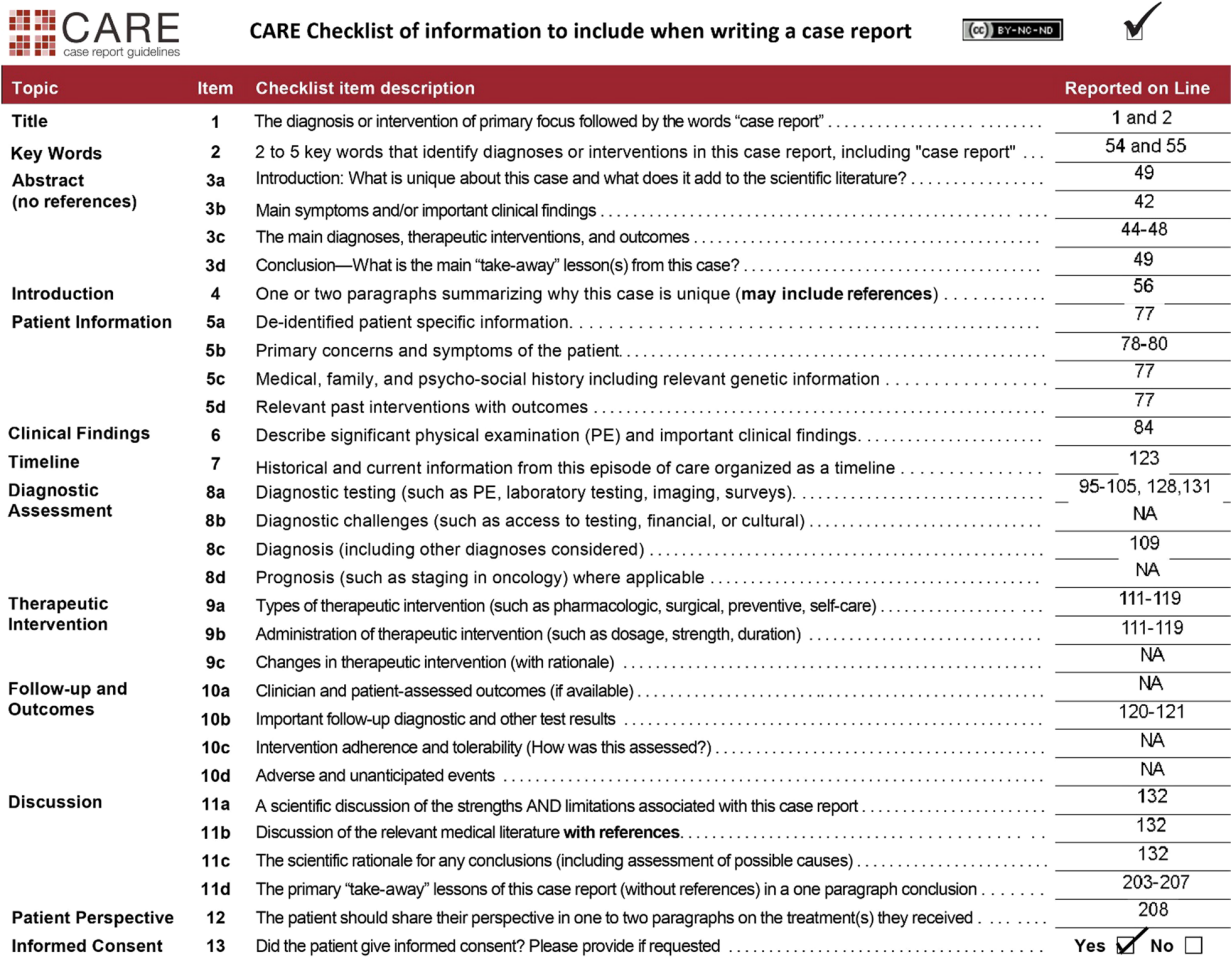
CARE checklist

## Conclusion

In conclusion, dengue fever presented in our case with a wide range of complications involving various organs, such as the brain, kidneys, liver, and myocardium. These complications ranged from encephalitis and seizures to acute kidney injury and myocarditis. It is important for healthcare professionals to be aware of the potential complications of dengue fever and to promptly diagnose and manage them in order to improve patient outcomes.

## Patient’s own perspective

The patient reported “As a young, healthy mother, I never expected to wind up in the intensive care unit struggling for my life. But that's exactly what happened when I contracted dengue fever. It all started with a high fever came on suddenly. I figured I had just caught a bug and would be feeling better soon, but my condition only seemed to get worse. Before long, I was experiencing changes in my mental status. When I arrived at the hospital, I was rushed to the emergency department for evaluation. The doctors told me that I had dengue fever and that it had caused complications, including brain inflammation. They immediately started me on treatment and transferred me to the intensive care unit. The next few days were a blur. I remember being hooked up to a lot of machines and feeling very weak and tired. My family was by my side, and the doctors and nurses were all very kind and compassionate, but I was in a lot of pain and was barely able to communicate. Eventually, I started to improve. I was transferred to a high dependency unit and was able to receive more targeted care. After a week, I was finally stable enough to be discharged from the hospital. Looking back, I am grateful to have survived this terrifying experience. But I also hope that others can learn from my story and take the necessary precautions to protect themselves from dengue fever. If you're traveling to an area where dengue is prevalent, be sure to use insect repellent and take other precautions to avoid mosquito bites. And if you do start to feel sick, don't wait to seek medical attention. Early diagnosis and treatment can make all the difference.”

## Data Availability

The data collected and analyzed during this case report are available upon request, subject to ethical and legal considerations. All data will be de-identified to protect the privacy of the patient.
